# Superpixel Segmentation Based on Grid Point Density Peak Clustering

**DOI:** 10.3390/s21196374

**Published:** 2021-09-24

**Authors:** Xianyi Chen, Xiafu Peng, Sun’an Wang

**Affiliations:** 1 Department of Automation, Xiamen University, Xiamen 361005, China; chenxy02@xmu.edu.cn; 2 School of Mechanical Engineering, Xi’an Jiaotong University, Xi’an 710049, China; sawang@mail.xjtu.edu.cn

**Keywords:** superpixel segmentation, density clustering, image preprocessing, computer vision

## Abstract

Superpixel segmentation is one of the key image preprocessing steps in object recognition and detection methods. However, the over-segmentation in the smoothly connected homogenous region in an image is the key problem. That would produce redundant complex jagged textures. In this paper, the density peak clustering will be used to reduce the redundant superpixels and highlight the primary textures and contours of the salient objects. Firstly, the grid pixels are extracted as feature points, and the density of each feature point will be defined. Secondly, the cluster centers are extracted with the density peaks. Finally, all the feature points will be clustered by the density peaks. The pixel blocks, which are obtained by the above steps, are superpixels. The method is carried out in the BSDS500 dataset, and the experimental results show that the Boundary Recall (BR) and Achievement Segmentation Accuracy (ASA) are 95.0% and 96.3%, respectively. In addition, the proposed method has better performance in efficiency (30 fps). The comparison experiments show that not only do the superpixel boundaries have good adhesion to the primary textures and contours of the salient objects, but they can also effectively reduce the redundant superpixels in the homogeneous region.

## 1. Introduction

Superpixel segmentation is often used as an important preprocessing method for image algorithms in different research fields, such as the image segmentation [1,2] and object recognition [3,4]. The superpixel segmentation is used to improve the diagnostic accuracy of medical images and the fruit and road recognition in agricultural automatic systems. It can improve the accuracy and efficiency of computer vision algorithms. Since the superpixel is used for image preprocessing by Ren [5], many well-known superpixel segmentation methods have been proposed by scholars. Color-based clustering is the most commonly used for superpixel segmentation, such as SLIC [6,7], LSC [8], ERS [9] and SEEK [10]. The regular superpixels are segmented by the SLIC and LSC, and the irregular superpixels are produced by the ERS and SEEK. Although the irregular superpixels make the following image algorithms more easily, the boundary adherence is worse than the ERS and SEEK. Their methods work well if the color gradient changes clearly between the different scenes in an image. In addition, a large number of redundant superpixels are produced in the smoothly connected region by those methods.

For improving the performance of the above methods, the DBSCAN [11,12] has been optimized in the boundary adherence by irregular superpixels. The sizes of the irregular superpixels in the smoothly connected region are larger by using the SEEDS [13] so that the redundant superpixels are reduced. Even though the methods are optimized, the redundant superpixels are abundant in superpixel images. Moreover, many useless boundaries, which cannot describe the primary texture and contour features are extracted.

**Figure 1:**
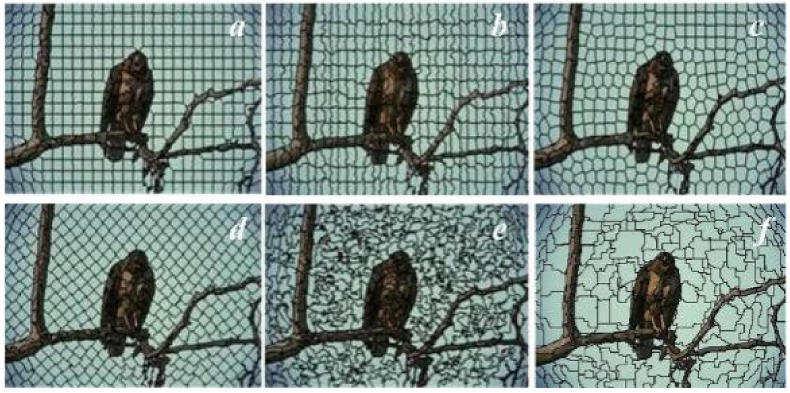
The six typical superpixel segmentation results. (**a**) SLIC [6], (**b**) PB [14], (**c**) LSC [8], (**d**) DBSCAN [11,12], (**e**) ERS [9], (**f**) SEEDS [13].

According to the superpixel boundary and shape, there are two main segmentation results as Figure 1 shows. (1) The superpixel shapes are more regular and evenly distributed in Figure 1a–d [11], but the superpixel boundaries cannot describe the texture and contour features well. (2) The boundary adherence in Figure 1e,f [9,13] are generally better than Figure 1a–d, but the boundaries and shapes are complex and very irregular. In addition, lots of redundant superpixels are produced by all of these methods.

Currently, since the superpixel segmentation is used as a preprocessing step in image processing, the classical and state-of-the-art superpixel methods focus primarily on how to improve the boundaries adherence and less on computation. Therefore, the over-segmentation and redundant superpixels in the smoothly connected region are often ignored. As a result, the complex and useless secondary texture and contour features in the image are extracted by superpixel boundaries. Those boundaries will increase the complexity and difficulty of image processing, such as image segmentation and image recognition.

This paper will focus on the superpixel segmentation whose superpixels can not only reduce the superpixel redundancy in the smoothly connected region, but the complex and useless background textures in an image can also be filtered so that the primary textures and contours of the salient objects can be extracted better. In order to meet the above requirements and successfully execute the described superpixel segmentation method, this paper is organized as follows.

First of all, the image is preprocessed, and the grid feature points are extracted. On this basis, the densities of all points are defined with color features in Section 2. Section 3 describes the superpixel segmentation method in detail. In Section 4, the superpixel segmentation experiments are carried out with the Berkeley database BSDS500. The effectiveness and superiority of the proposed method will be discussed in Section 5. Finally, Section 6 concludes the study.

## 2. Image Preprocessing

### 2.1. The Feature Point and Color Feature Extraction

The color and brightness features are always different among the different scenes in images. Generally, the contour and texture features can be extracted by the boundary lines between two adjacent scenes or objects in an image. Therefore, the color and brightness features can be used in the pixel clustering method. After that, the pixel blocks with different shapes and sizes will be obtained. The boundaries of the pixel blocks are extracted to use for superpixel segmentation.

Generally, the RGB color space is difficult to extract the brightness feature features; therefore, the RGB color space will be converted into the Lab color space, which not only includes the brightness features, but also has a wide color gamut to improve the accuracy of the color feature extraction. The conversion matrix of the RGB to Lab color space is shown in the following Equations (1)–(3).

(1) $$\left[\begin{array}{c} X\\ Y\\ Z \end{array}\right]=\left[\begin{array}{ccc} 0.4124 & 0.3576 & 0.1805\\ 0.2126 & 0.7152 & 0.0722\\ 0.0193 & 0.1192 & 0.9505 \end{array}\right]\left[\begin{array}{c} R\\ G\\ B \end{array}\right]$$

(2) $$\left\{\begin{array}{l} L=116f(Y/Y_{n})-16\\ a=500[f(X/X_{n})-f(Y/Y_{n})]\\ b=200[f(Y/Y_{n})-f(Z/Z_{n})] \end{array}\right.$$

(3) $$f(t)=\left\{\begin{array}{l} t^{1/3}\\ \frac{1}{3}{\left(\frac{29}{3}\right)}^{2}t+\frac{4}{29} \end{array}\right.\begin{array}{c} t>{\left(\frac{6}{29}\right)}^{3}\\ t\leq {\left(\frac{6}{29}\right)}^{3} \end{array}$$

The values of *X_n_*, *Y_n_* and *Z_n_* are 0.9505, land 1.089, respectively. The image size is 320 × 240 (width × height) in this paper. The parameters *R*, *G*, *B* and *L*, *a*, *b* are the pixel values in RGB and Lab color space, respectively.

In this paper, the pixels which meet the Formula (4) will be extracted as initial feature points. The parameters *h*, *w* and *r*, *c* are the row and column coordinates of the feature points and pixels in the image. *R* is the interval between any two adjacent feature points.

(4) $$\left\{\begin{array}{l} h=Rr\\ w=Rc \end{array}\right.\begin{array}{c} r=0,1,2,3,\cdots ,240/R\\ c=0,1,2,3,\cdots ,320/R \end{array}$$

The values of the color and brightness of the feature points can be computed with the Formulas (5). The parameters $\bar{L}$, $\bar{a}$, and $\bar{b}$ are also the feature parameters of the points. The color and brightness features will be used in the cluster.

(5) $$\bar{L}=\frac{\sum_{i=h-R/2}^{h+R/2}\sum_{j=w-R/2}^{w+R/2}L_{i,j}}{R^{2}},\bar{a}=\frac{\sum_{i=h-R/2}^{h+R/2}\sum_{j=w-R/2}^{w+R/2}a_{i,j}}{R^{2}},\bar{b}=\frac{\sum_{i=h-R/2}^{h+R/2}\sum_{j=w-R/2}^{w+R/2}b_{i,j}}{R^{2}}$$

**Figure 2:**
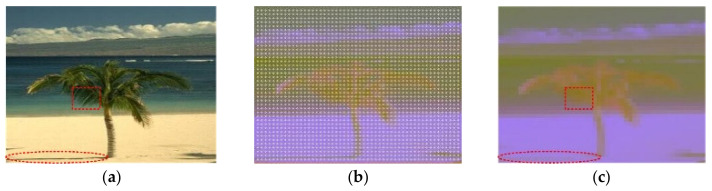
Image preprocessing. (**a**) Original image, (**b**) Lab image and the grid feature points, (**c**) the smoothed and filtered image.

*L_i,j_*, *a_i,j_* and *b_i,j_* are the pixel values in the Lab color space, and *i* and *j* are the row and column coordinates of the pixels, which is an *R* × *R* region around the feature points. In fact, the Formulas (5) play the role of the image mean filtering, and the filter window is *R* × *R*. The filtering can effectively smooth the image, reduce the complex and redundant texture features and highlight the primary contour features. According to Figure 2a,c, the spiculate margin of the leaves in the rectangular box and the shadows of the trunk in the ellipse box are smoothed and filtered. The Lab color space image and the feature points are shown in Figure 2b. The number of feature points is *N* = 320 × 240/*R*^2^. (*R* = 6 as an example in this section).

### 2.2. The Feature Point Density

**Figure 3:**
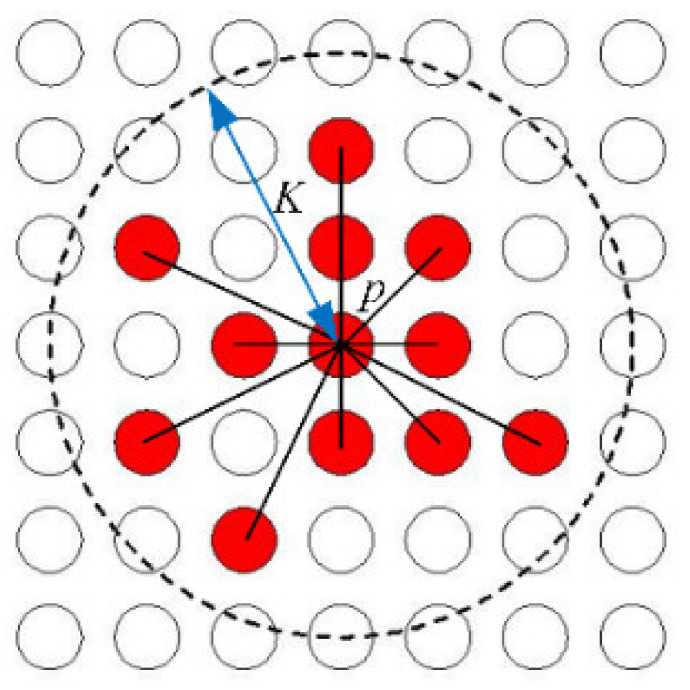
The density schematic diagram of the point *p*.

The density-based clustering method is used in this paper [15]. The density schematic diagram of each point is shown in Figure 3. The red center point is the point *p* whose density will be calculated. The other red points in the *K*-neighborhood are the points whose color and brightness features are similar to *p*. The density value of *p* is the sum of the red feature points that in the *K*-neighborhood. Therefore, the greater the point density, the more similar the color in its neighborhood.

The density *ρ* of the point *p*(*h*, *w*) is calculated by the Formulas (6) and (7). The Formula (6) is used for extracting the feature points that the color and brightness features are similar to *p*(*h*, *w*). The Formula (7) is used for extracting the feature points which are in the *K*-neighborhood.

(6) $$\left\{\begin{array}{c} \rho =\sum_{\sqrt{{\left(i-h\right)}^{2}+{\left(j-w\right)}^{2}}\leq K}\chi (\sqrt{{\left({\bar{L}}_{ij}-{\bar{L}}_{hw}\right)}^{2}+{\left({\bar{a}}_{ij}-{\bar{a}}_{hw}\right)}^{2}+{\left({\bar{b}}_{ij}-{\bar{b}}_{hw}\right)}^{2}})\\ \chi (x)=\left\{\begin{array}{l} 0,x>\psi \\ 1,x\leq \psi \end{array}\right. \end{array}\right.$$

(7) $$D=\sqrt{{\left(i-h\right)}^{2}+{\left(j-w\right)}^{2}}$$

The parameters *h* and *w*, *i* and *j* are the coordinates of the center point *p*(*h*, *w*) and the other red point *p*(*i*, *j*), respectively. The symbol *ψ* is the color threshold parameter. The variable *D* describes the distance from the center point to any other feature point. Figure 3 shows that the number of the red feature points is 12, which means that the density of *p* is 12 and that *ρ* = 12.

## 3. Superpixel Segmentation with Density Peak-Based Clustering

In this section, there are two rules about the color and brightness features for the superpixel segmentation method.

**Figure 4:**
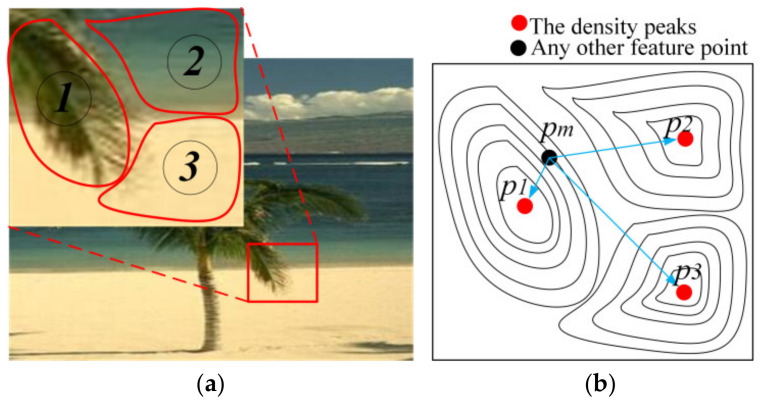
The probability distribution of the points. (**a**) The superpixel segmentation schematic diagram. (**b**) The density peaks and the contour maps are used to describe the density distribution of the other points.

(1) The more similar the color and brightness features between two points, the more similar the densities of them. In this case, the two feature points are easier to be segmented into the same superpixel.

(2) If the color and brightness features are all different among the neighborhood points, they will be segmented into different superpixels even if the densities of them are similar. For example, as Figure 4a shows, the regions of the foliage, the sea and the beach have obvious differences in color and brightness features, so that the three regions would be probability segmented into three superpixels, which are marked by ①, ② and ③.

There must be at least one feature point whose density is higher than the neighborhood points; for example, the red points in Figure 4b are defined as density peaks in this paper. The density peaks will be probability defined as the cluster center. According to the densities, the other points could be distributed among the density peaks, and they can be described as the contour maps. For example, *p_m_* is one of many other feature points, and if the density of *p_m_* is close to *p*_1_ and the distance between them is also closed, the point *p_m_* and *p*_1_ will be segmented in the same superpixel with high probability.

First of all, the densities of all the feature points must be calculated by the Formulas (6) and (7), and the results are stored in a data set {*ρ_m_* | 0 ≤ *m* ≤ *N*}, *m* is the sequence number of the points and *N* is the total number of the feature points. According to the densities, all the points are sorted in descending order. {*o_m_* | 0 ≤ *m* ≤ *N*} is the result after rearrangement and *o_m_* is the feature point. *D_mn_* is the distance between any two points *o_m_* and *o_n_. D_mn_* can be calculated by the Formula (7).

In order to cluster the points, the membership grade between any two points must be obtained. The membership grade parameter *δ_m_* can be obtained by Formula (8).

(8) $$\delta_{m}=\left\{\begin{array}{c} \underset{m>0,n<m}{\min}\{D_{mn}\}\\ \underset{m=0,n>0}{\max\{\delta_{n}\}} \end{array}\right.$$

If *m* > 0 and *n* < *m*, *δ_m_* is the minimum distance between *o_m_* and *o_n_*. Actually, *δ_m_* means the probability that *o_m_* and *o_n_* are in the same cluster or not. If *m* = 0, *δ*_0_ is the maximum of all *δ*. The parameters *δ_m_* and *ρ_m_* are used to extract cluster centers. If *δ_m_* and *ρ_m_* both are relatively large, it means that the point *o_m_* would be a density peak and it will be defined as the cluster center with great probability. If *m* = 0, *o*_0_ must be a cluster center.

To determine whether the point *o_m_* is a cluster center or not, the *δ_m_* and *ρ_m_* should first be normalized, and the values fall within the range of [0, 1]. The normalization formulas are shown as follows.

(9) $$\rho_{m}^{\prime }=\frac{\rho_{m}-\rho_{\min}}{\rho_{\max}-\rho_{\min}},\;\delta_{m}^{\prime }=\frac{\delta_{m}-\delta_{\min}}{\delta_{\max}-\delta_{\min}}$$

**Figure 5:**
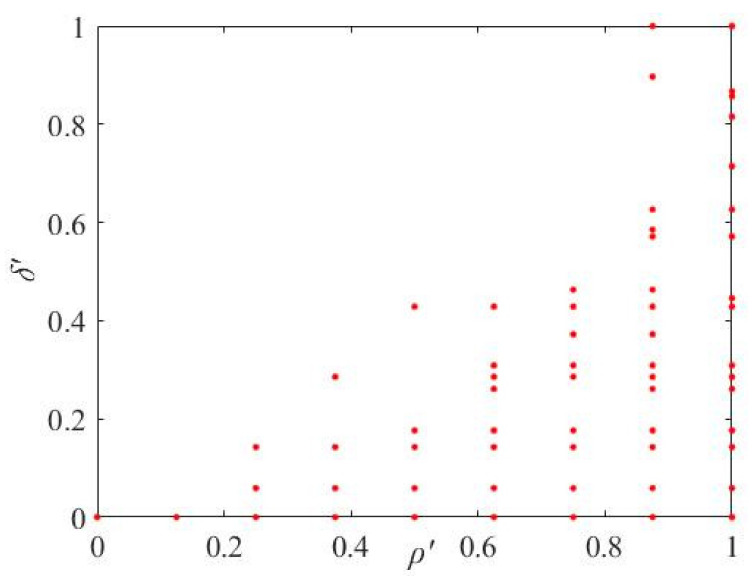
The distribution of all the feature points in the *ρ*′–*δ*′ coordinate system.

*Ρ*′ and *δ*′ are the normalized parameters, *ρ*_max_ and *ρ*_min_ are the maximum and minimum values of *ρ*, *δ*_max_ and *δ*_max_ are the maximum and minimum values of *δ*. In order to analyze how to extract the cluster center, Figure 2 is used as an example. The neighborhood parameter *K* and the threshold parameter *ψ* are assigned firstly, with *K* = 1.3*R* and *ψ* = 13 as an example. Then the *ρ*′–*δ*′ coordinate system will show the distribution of all the feature points in Figure 5.

It can be seen from Figure 5 that the larger the *ρ*′ and *δ*′ of the points are, the more likely it is that the points are distributed in the upper right part of the coordinate system. In other words, the points in the upper right part are the candidates for cluster centers. For extracting the cluster centers from the points, the following two Equations (10) or (11) are used to determine which ones are the cluster centers in this paper.

(10) $$\delta^{\prime }=\frac{a}{\rho^{\prime }}$$

(11) $$\lambda =\rho^{\prime }\delta^{\prime }$$

**Figure 6:**
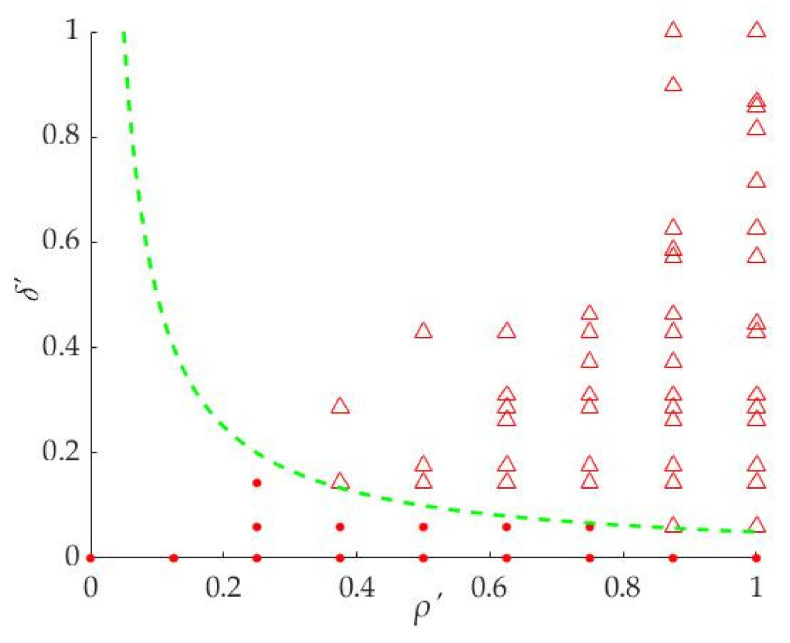
Extracting the cluster centers by inverse function. The triangle symbols are the center points and the red dots are the other points.

The Equation (10) is an inverse function and its function curve divides the points into two parts, the upper right part and the lower left part. We take *a* = 0.005 as an example for analysis in this section. The points ‘Δ’ will be the cluster centers as shown in Figure 6 and the set of the cluster centers is ***C***_M_, and M is the total number of cluster centers. In this step, the curve of the function can be changed by the parameter *a*, so that the different cluster centers can be extracted to meet the requirement of the superpixel segmentation algorithm. The method can extract the cluster centers well, but it is difficult to predict the number of the cluster centers.

**Figure 7:**
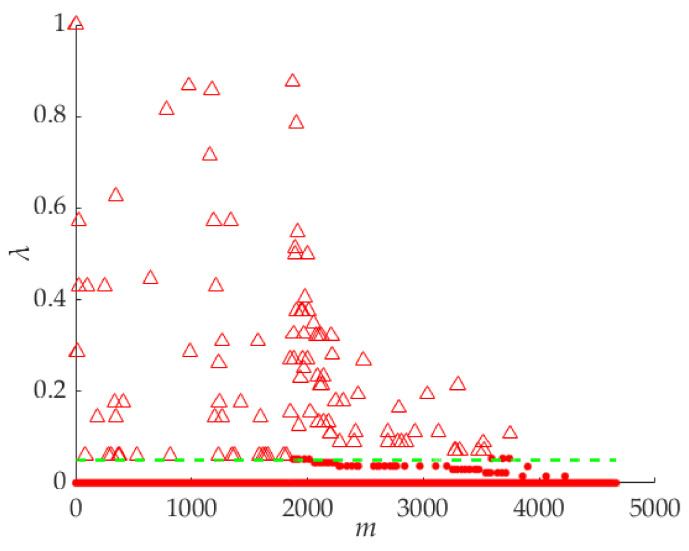
Extracting the cluster centers by *λ.* The triangle symbols are the center points, and the red dots are the other points.

The parameter *λ* in Equation (11) is a comprehensive parameter of the *ρ*′ and *δ*′. The greater the value of *λ*, the greater the probability that the points are the cluster centers. All the points are shown in the *m*–*λ* coordinate system in Figure 7. The values of the *m*-axis are the serial number of all the points. We take *λ* = 0.005, for example, and the points on the upside of the function curve are the cluster centers.

Sometimes the number of the cluster centers must be obtained before superpixel segmentation. For producing a specified number of cluster centers, the parameter λ will be sorted in descending order *λ*_0_ > *λ*_1_ > *λ*_2_ > … > *λ_m_* *>* … > *λ*_N−1_ > *λ*_N_. *N* is the total number of the feature points, *m* is the serial number of the feature points. If the specified number is *M*, the points whose *λ_m_* are meet the condition {*λ_m_* | 0 ≤ *m* ≤ *M*} will be the cluster centers, and the number of the cluster centers is M.

After obtaining the cluster centers, the other points *o_m_* would be clustered to one of them. According to the Formula (8), if *δ_m_* = D*_mn_*, it means that the points *o_m_* and *o_n_* would be in the same cluster, and they will be expressed by *o_m_*→*o_n_* in this paper. According to whether *o_n_* is a cluster center or not, there are two cases about how to cluster the point *o_m_*.

Case 1, if *o_n_* is a cluster center, *o_m_* will be a member of the cluster which include the *o_n_*;

Case 2, if *o_n_* is not a clustered center, iterative and recurrence such as *o_m_*→*o_n_*, *o_n_*→*o_h_*, *o_h_*→*o_i_*, *o_i_*→*o_j_*, …, until the cluster center is found. In this case, it is easy to deduce that *o_m_*→*o_j_*_,_ and if the point *o_j_* is a cluster center, the point *o_m_* will be a member of the cluster in which the *o_j_* is cluster center.

According to the above method, all the points *o_m_* would be clustered, and the ensemble of the clusters are represented by the set *S*. The key steps of the clustering method are given as follows. Algorithm 1 [15] The key steps of the Density Peak-Based Clustering.
**Algorithm 1.** Density Peak-Based Clustering**Input:** The set of the feature points ***O****_n_*;   The set of the parameters ***δ****_n_*;   The set of the parameters ***λ****_n_*;**Output:** Ensemble of clusters ***S***;1: Extracting the set of cluster centers from the feature points ***O****_n_* with the help of the parameters ***δ****_n_* or ***λ****_n_*. The set of the cluster center is ***C***_M_;2:   **for** each *i*∈[0, *N*] **do** 3:       Iterative and recurrence formulas *o_i_*→*o_h_*, *o_h_*→*o_j_*, …, *o_k_*→*o_c_*,until the cluster center *o_c_* is found; 4:       *o_i_*→*o_c_*;5:   **end for**6:   **for** each *i*∈[0,*M*] **do**7:    **for** each *j*∈[0, *N*] **do**8:       **if** the point *o_j_* meet the condition *o_j_*→*c_i_* is true **then**9:          ***S****_i_*= ***S****_i_*∪*o_j_*;10:       **end if**11:    **end for**12:  **end for**13:  **return *S***;

## 4. Superpixel Segmentation Experiment Results

**Figure 8:**
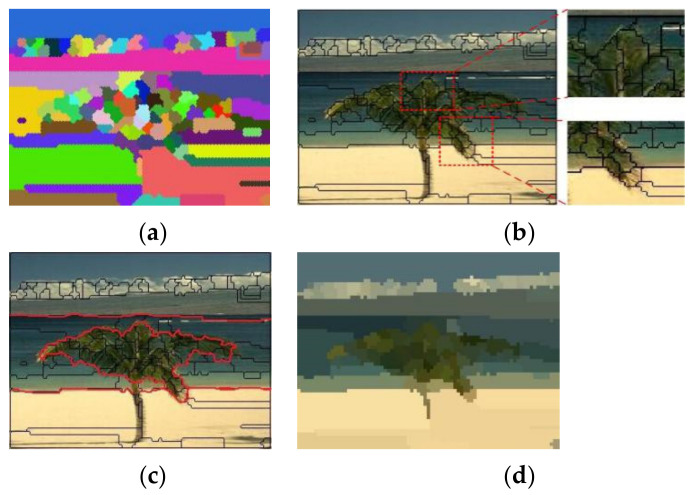
The results of the superpixel segmentation experiment. (**a**) The color blocks represent the different superpixels, (**b**) the black lines show the superpixel boundaries, (**c**) The red segmentation lines between the different salient regions, (**d**) The superpixel image.

The clusters in Section 3 are actually the pixel blocks, and they are used for superpixel segmentation. The image in Figure 2a is used as an example for the superpixel segmentation experiment. According to the method in Section 3, the initial value should be assigned to the parameters *R*, *K*, *ψ* and *a*; for example, if *R* = 6, *K* = 1.3R, *ψ* = 13 and *a* = 0.005. The results of the cluster are shown in Figure 8a with the color blocks. Actually, the blocks are the superpixels. Figure 8b shows the boundaries of the superpixels, and the primary textures and contours of the objects are drawn very well by the boundaries. In addition, the skyline, the shoreline and the outline of the tree can be drawn with the red lines that are drawn by concatenation of superpixel boundaries in Figure 8c. The image can be segmented into four regions by the red lines representing the beach, the sea, the sky and the tree. Figure 8d is a superpixel image, and it is clear that the image can not only draw the salient objects well, such as the beach, the sea, the sky and the tree, but can also reduce the redundant complex textures of the image, such as the spiculate margin of the leaves.

It is easy to conclude that the results of superpixel segmentation mainly depend on these parameters *R*, *K*, *ψ*, *a* and *λ*. Generally, if there is a good segmentation result with a set of parameter values such as *K* = 1.3R, *ψ* = 13, *a* = 0.005, *λ* = 0.005, the different superpixel segmentation results can be obtained by changing the value of the parameter R without other parameters changed.

**Figure 9:**
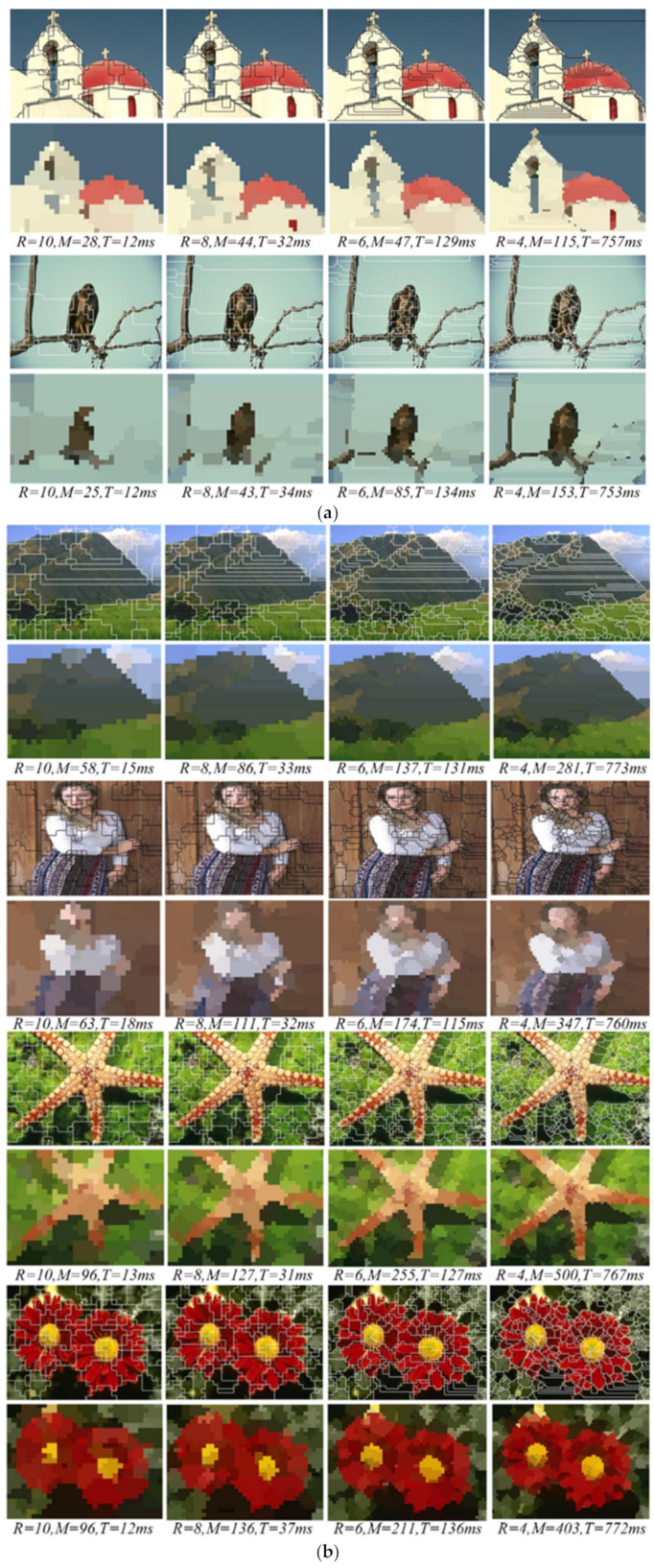
The superpixel segmentation results. The segmentation results are obtained with the different *R*. (**a**) The images which include the large smoothly connected homogenous regions are segmented by fewer and larger superpixels. (**b**) The images which include the complex textures and contours are segmented by more and smaller superpixels.

In order to analyze the superpixel segmentation results with the proposed method, the Berkeley database BSDS500 is used in this paper, and the sample images are resized to 320 × 240. The experiments are performed when *R* = 4, 6, 8 and 10, respectively. The superpixel segmentation results are shown in Figure 9. It can be seen from the segmentation results that the smaller the *R* is, the better the boundaries adherence is, and the clearer the textures and boundaries of the objects. It can be found that regardless of the value of *R*, the superpixels can depict the salient objects every well, such as the eagle, starfish, flowers and people. (The method was tested on a computer with an Intel i5 @ 2.5 GHz processor with 4 GB RAM).

The texture complexity of different images is often different, so that the number and the size of superpixels are different from one image to another. As Figure 9a shows, the textures and contours of the building, bird and branches are relatively simple, and there are large smoothly connected homogenous regions, so that the satisfactory segmentation results can be obtained with fewer and larger superpixels. However, in Figure 9b, more and smaller superpixels must be segmented to extract the complex textures and contours of the mountain, people, starfish and flowers. Experiments showed that small and larger value of the parameter *R* can be taken in Figure 9a,b respectively. Therefore, the appropriate values of the parameter *R* can be taken for producing satisfactory superpixels according to the texture complexity. Furthermore, even if the value of the parameter R remains the same, the numbers and the sizes of the superpixels are always different so that the segmentation method can adapt to the different images. The segmentation results clearly show that the method can not only reduce the redundant superpixels in smoothly connected homogenous regions, but the textures and contours of the salient objects in the image can also be depicted very well by the superpixel boundaries. In addition, the contours between different objects in the image are extracted well by the boundaries. The boundary recalls of the salient objects are shown in Table 1.

It can be seen from Table 1 that the smaller value of the parameter *R*, the better Boundary Recall, and that means the better boundary adherence. Otherwise, there is not much difference in Boundary Recall in the superpixel segmentation in different images with the same value of the parameter *R*. This means that the method could work well whether the texture features are simple or complex in different images. Therefore, it is further proved that the proposed method has good adjustability.

**Figure 10:**
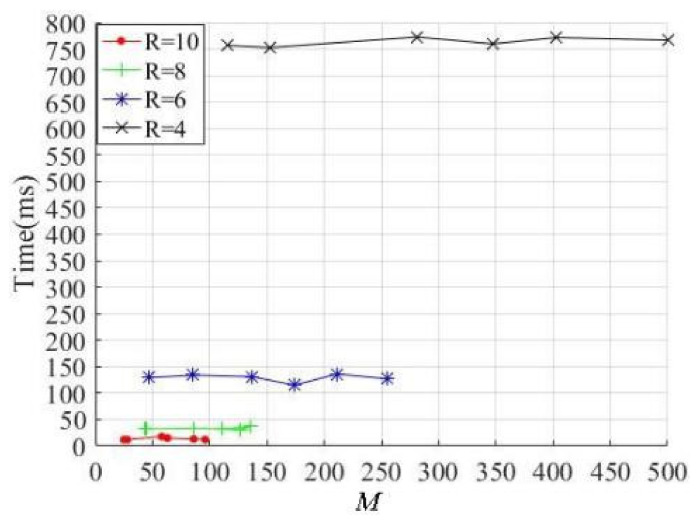
The superpixel segmentation time-consuming with different values of *R*.

In the process of superpixel segmentation, the algorithm efficiency must be considered. The relationship between the superpixel number *M* and the segmentation efficiency are shown in Figure 10.

Figure 10 shows that the efficiencies are determined with the parameters *R* = 4, 6, 8 and 10, respectively. Obviously, the larger the parameter values, the more efficient the method. Although the superpixel numbers increase with the increase in texture complexity, the efficiency is almost constant when the value of the parameter *R* remains unchanged. Therefore, it can be concluded that the method efficiency mainly depends on the parameter *R*, and it is almost independent of the number of superpixels. The segmentation results in Figure 9 show that if the *R* = 8 and 10, the primary texture and contour features of the salient objects in an image can be extracted well, and the average times are 13.8 ms and 33.2 ms, respectively. This means that the proposed method has good efficiency under the condition that the segmentation results are satisfactory.

## 5. Discussion

**Figure 11:**
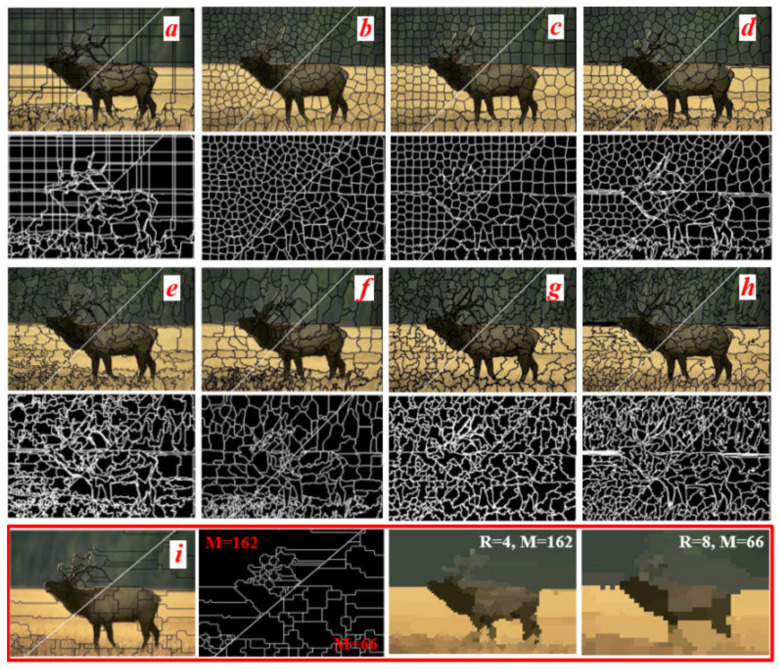
The segmentation results comparison of the different superpixel methods. (**a**) Lattice [17], (**b**) Ncuts [5], (**c**) SLIC [6], (**d**) LSC [8], (**e**) SEEDS [13], (**f**) EneOpt1 [18], (**g**) quick shift [19], (**h**) ERS [9], (**i**) ours.

According to the shape and size of the superpixels, the superpixel methods can be divided into two cases. For example, the typical and well-known methods, such as the Lattice, ERS, SLIC and LSC, could produce regular superpixels. The irregular superpixels with sawtooth boundaries would be obtained by methods such as SEEDS, EneOpt1, quick shift and ERS. The segmentation results of them [8,16] are shown in Figure 11a–h. The number of the superpixels in the upper left and lower right parts in image are approximately 400 and 200, respectively. In order to obtain the similar segmentation results, we take *R* = 4 and 8 for segmentation, and the results of the proposed method are shown in Figure 11i. The numbers of superpixels are *M* = 162 and *M* = 66 in the upper left and lower right parts.

Generally, the primary textures and contours of the image are the features that we care about in superpixel segmentation. Those are benefits to improve the accuracy of image segmentation and object recognition. According to the comparison results, although the more regular superpixels can be obtained in Figure 11a–d and they can help to reduce the complexity of the image recognition methods, a large number of redundant superpixels are produced in the smoothly connected homogenous regions. The texture and contour features are extracted very well, and the boundaries have good adhesion in Figure 11e–h. That will help for extracting the target objects in the image more accurately. However, not only are most of the secondary textures extracted, which does not help with object recognition, but the extremely irregular and redundant superpixels are also produced in the smoothly connected homogenous regions. Normally, that will improve the complexity and reduce the efficiency of the image recognition algorithm. Compared to Figure 11a–h, the smoothly connected homogenous regions are segmented by fewer superpixels, and the superpixels are sketched by fewer jagged boundaries in Figure 11i. In addition, the primary textures and contours of the salient objects in the image can be extracted accurately by the proposed method. In comparison with the other methods, the proposed method can greatly reduce the redundant superpixels, and that could improve the efficiency of the superpixel algorithm.

**Figure 12:**
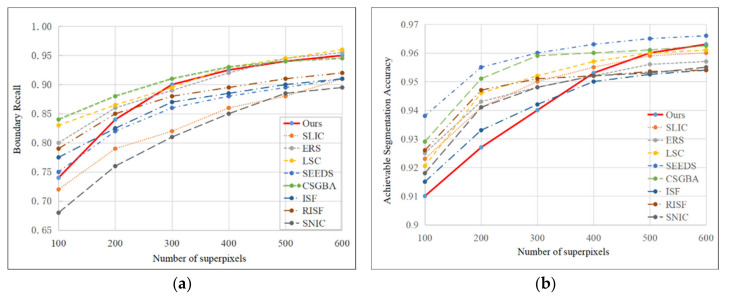
(**a**,**b**) Boundary Recall and Achievement Segmentation Accuracy of the superpixel methods on the BSDS500 dataset. The SEEDS, SLIC, ERS and LSC are examples of the classical methods. CSGBA [20], ISF [21], RISF [22] and SNIC [23] are the examples of the state-of-the-art methods.

Moreover, with the decrease in R and the increase in the number of superpixels, the superpixel boundaries will become smoother, and the boundary adherence will be better as well. In order to analyze the effectiveness of the proposed method, the Boundary Recall (BR) and Achievement Segmentation Accuracy (ASA) of ours and the state-of-the-art superpixel segmentation methods are shown in Figure 12.

A comparison of each superpixel segmentation method is proposed in Figure 12. It is clear that the BR and ASA of the proposed method have good results. Specifically, the BR can achieve satisfactory results with a smaller number of superpixels (*M* = 300), and it is more than 90%. That is better than most of the classical and the state-of-the-art superpixel segmentation methods and it is 95.0% when *M* = 600. If the number of superpixels is too small, the results of the ASA are inferior to other methods. However, as the number M increases, the results of the ASA improve quickly. The ASA is 96.3% when *M* = 600, and it is better than most of the methods.

It is well-known that the efficiency of a superpixel method is very important for real-time image processing. The segmentation results in Figure 9 and Figure 11 show that the primary texture and contour features of the salient objects in the image can be extracted well by the superpixels, in which the parameter *R* = 8. In addition, the superpixel boundaries have good adhesion to the textures and contours. Therefore, the efficiency of the proposed method would be analyzed by *R* = 8. To be clearer, the numeric values of the metrics are listed when the number of superpixels *M* ≈ 400. The computational time and BR of the segmentation methods are summarized, and they are shown in Table 2.

It can be seen from Table 2 that the efficiency of the proposed method is superior to the classical and state-of-the-art methods. The speed of the superpixel segmentation is nearly 30 fps, and it indicates that the method has better real-time performance. Moreover, the BR is 92.5% with the same condition, and it is better than most of the classical and state-of-the-art methods. This means that the proposed method has good comprehensive performance both in efficiency and accuracy.

## 6. Conclusions

The proposed superpixel segmentation method in this paper is based on the density peak of the feature points. The shape and size of the superpixels depend on the density values and their distribution in the image. The density values are larger in the smoothly connected homogenous regions. Therefore, the larger size of superpixels would be produced and the redundant superpixels can be reduced. Moreover, the secondary complex texture features would be effectively filtered and smoothed in the superpixel images. The superpixel boundaries also have good adhesion to the primary textures and contours. That allows the boundaries of the salient objects in an image to be described well, which would be helpful for the following image process, such as object segmentation and image recognition. On the premise of the satisfying results of superpixel segmentation, the efficiency of our method is almost the best among the classical and state-of-the-art methods. In conclusion, the proposed method has good comprehensive performance both in efficiency and accuracy, especially the images which include the salient objects and lots of smoothly connected homogenous regions.

In future work, the parameters *a* and *λ* will be researched to optimize the proposed method so that the algorithm efficiency and accuracy can be improved with the small value of parameter R.

## Figures and Tables

**Table 1:** Boundary Recall (%).

Object	*R*			
	10	8	6	4
building	69	82	88	92
eagle	73	82	87	93
mountain	71	81	89	94
people	68	72	80	91
starfish	77	87	89	96
flower	78	89	92	95

**Table 2:** The comparison of the efficiency and BR (*M* ≈ 400).

Methods	Average Time per Image(s)	Boundary Recall (%)
SEEDS	0.059	88.6
SLIC	0.087	86.1
ERS	0.798	92.0
LSC	0.255	93.2
CSGBA	0.146	93.0
ISF	0.151	88.5
RISF	0.082	89.4
SNIC	0.064	88.5
Ours	0.033	92.5

## Data Availability

Not applicable.
